# The Spatial Pattern and Interactions of Woody Plants on the Temperate Savanna of Inner Mongolia, China: The Effects of Alternating Seasonal Grazing-Mowing Regimes

**DOI:** 10.1371/journal.pone.0133277

**Published:** 2015-07-21

**Authors:** Xiao Wang, Bo Zhang, Kebin Zhang, Jinxing Zhou, Bilal Ahmad

**Affiliations:** Soil and Water Conservation, Beijing Forestry University, Haidian District, Beijing, China; Technical University in Zvolen, SLOVAKIA

## Abstract

*Ulmus pumila* tree-dominated temperate savanna, which is distributed widely throughout the forest-steppe ecotone on the Mongolian Plateau, is a relatively stable woody-herbaceous complex ecosystem in northern China. Relatively more attention has been paid to the degradation of typical steppe areas, whereas less focus has been placed on the succession of this typical temperate savanna under the present management regime. In this study, we established 3 sample plots 100 m×100 m in size along a gradient of fixed distances from one herder’s stationary site and then surveyed all the woody plants in these plots. A spatial point pattern analysis was employed to clarify the spatial distribution and interaction of these woody plants. The results indicated that old *U*. *pumila* trees (DBH ≥ 20 cm) showed a random distribution and that medium *U*. *pumila* trees (5 cm ≤ DBH < 20 cm) showed an aggregated distribution at a smaller scale and a random distribution at a larger scale; few or no juvenile trees (DBH < 5 cm) were present, and seedlings (without DBH) formed aggregations in all 3 plots. These findings can be explained by an alternate seasonal grazing-mowing regime (exclosure in summer, mowing in autumn and grazing in winter and spring); the shrubs in all 3 plots exist along a grazing gradient that harbors xerophytic and mesophytic shrubs. Of these shrubs, xerophytic shrubs show significant aggregation at a smaller scale (0-5.5 m), whereas mesophytic shrubs show significant aggregation at a larger scale (0-25 m), which may be the result of the dual effects of grazing pressure and climate change. Medium trees and seedlings significantly facilitate the distributions of xerophytic shrubs and compete significantly with mesophytic shrubs due to differences in water use strategies. We conclude that the implementation of an alternative grazing-mowing regime results in xerophytic shrub encroachment or existence, breaking the chain of normal succession in a *U*. *pumila* tree community in this typical temperate savanna ecosystem. This might eventually result in the degradation of the original tree-dominated savanna to a xerophytic shrub-dominated savanna.

## Introduction

In the early 1980s, the Livestock and Rangeland Double-Contract Responsibility System (LRDCRS) was implemented in the pastoral regions of Northern China, with strong expectations of increasing livestock productivity and rehabilitating degraded rangeland[1, 2]. This measure was followed by the establishment of fixed and exclusive resource boundaries and the adoption of alternating seasonal grazing (winter and spring) and mowing (autumn) regimes as a commonly accepted management tool[1, 3]. However, some research shows that rangeland degradation was never halted[4–7]. This continued degradation has been attributed to climatic change, such as long-term and high frequent drought, or human activities, such as overgrazing and overexploitation [8–10]. Shrub encroachment into grassland has also been considered an obvious indicator of rangeland degradation in the pastoral regions of northern China[11, 12]. As a common tree species distributed widely throughout the forest-steppe ecotone on the Mongolian Plateau[13, 14], in association with grasses, *Ulmus pumila* trees form a stable savanna-like woody-herbaceous complex ecosystem in the Horqin Sandy Land, the Otindag Sandy Land and the Hulunbeier Sandy Land of northern China[15, 16]. Sparse *U*. *pumila* trees within the savanna have ecological significance in sand stabilization and small-habitat provision for livestock[17]. However, although it represents an important consideration for the steppe in this region, the degradation of this temperate savanna, especially the sparse *U*. *pumila* tree pattern in recent years, has attracted little attention from the public or scientists [18, 19]. A small number of studies have shown that, compared with other species, *U*. *pumila* has faced a major regeneration challenge [20–23]. *U*. *pumila* seedlings often suffer from severe water stress during dry summers is caused by repeated cycles of drying in the upper soil layers [24, 25]. Wang et al. [22] noted that more than 90% of the current-year seedlings in fenced plots died because of their vulnerability to drought. Jiang et al. [23] discussed the effect of vegetation cover on the recruitment of *U*. *pumila*, finding that the highest initial seed density was found under the highest vegetation cover.

Spatial pattern analysis is an important method for studying the interactions and relationships among different plant populations and their environments [26, 27]. The analysis of a species’ spatial pattern will help us to understand both the ecological process that forms the pattern (such as seed dispersal, intra- and inter-specific competition, interference, and environmental heterogeneity) and the ecophysiological traits of the plant species, including the relationship between these plants and the environment[28–30]. The spatial pattern of species and the spatial correlation between species have a significant impact on growth, reproduction, death, resource utilization and gap formation among species [29, 31]. Recently, a spatial pattern analysis method has been used for clarifying the vegetation degradation processes underlying the individual pattern in semi-arid and arid areas [32–35].

In this study, we selected 3 fixed sample plots 100 m×100 m in size along a predetermined grazing gradient (distance from the herder's stationary site), and the spatial pattern of the individual woody plants (including *U*. *pumila trees* and shrubs) and interactions among these woody plants were analyzed using the spatial point pattern method. Two key points are discussed: (1) whether an alternating seasonal grazing-mowing regime affects the pattern of elm trees and shrub occurrence or presence, and (2) whether alternating seasonal grazing-mowing management reduces recruitment of the *U*. *pumila* trees. This study will be helpful for a better understanding of the degradation processes affecting a *U*. *pumila*-dominated savanna.

## Materials and Methods

### Ethics Statement

The study site is located in the Otindag Sandy Land. It is administered by the Sanggendalai township of the Xilinguole League, Inner Mongolia (115°16'E, 42°50'N). The study was conductedon private land. We paid the owner of the land for permission to conduct the study on this site. No rare or endangered wild animals or plants were collected in this experiment. Permit restrictions also required the experiment to be conducted without the use of open flame to prevent forest fires and without the cutting of tall trees to protect the forest ecosystem from damage. In addition, the samples consisted of common tree species and shrubs, so sampling had no direct impact on vertebrate survival. This experiment did not use wild animals or plants as research objects and did not pose a threat to the environment.

### Study site

**Fig 1:**
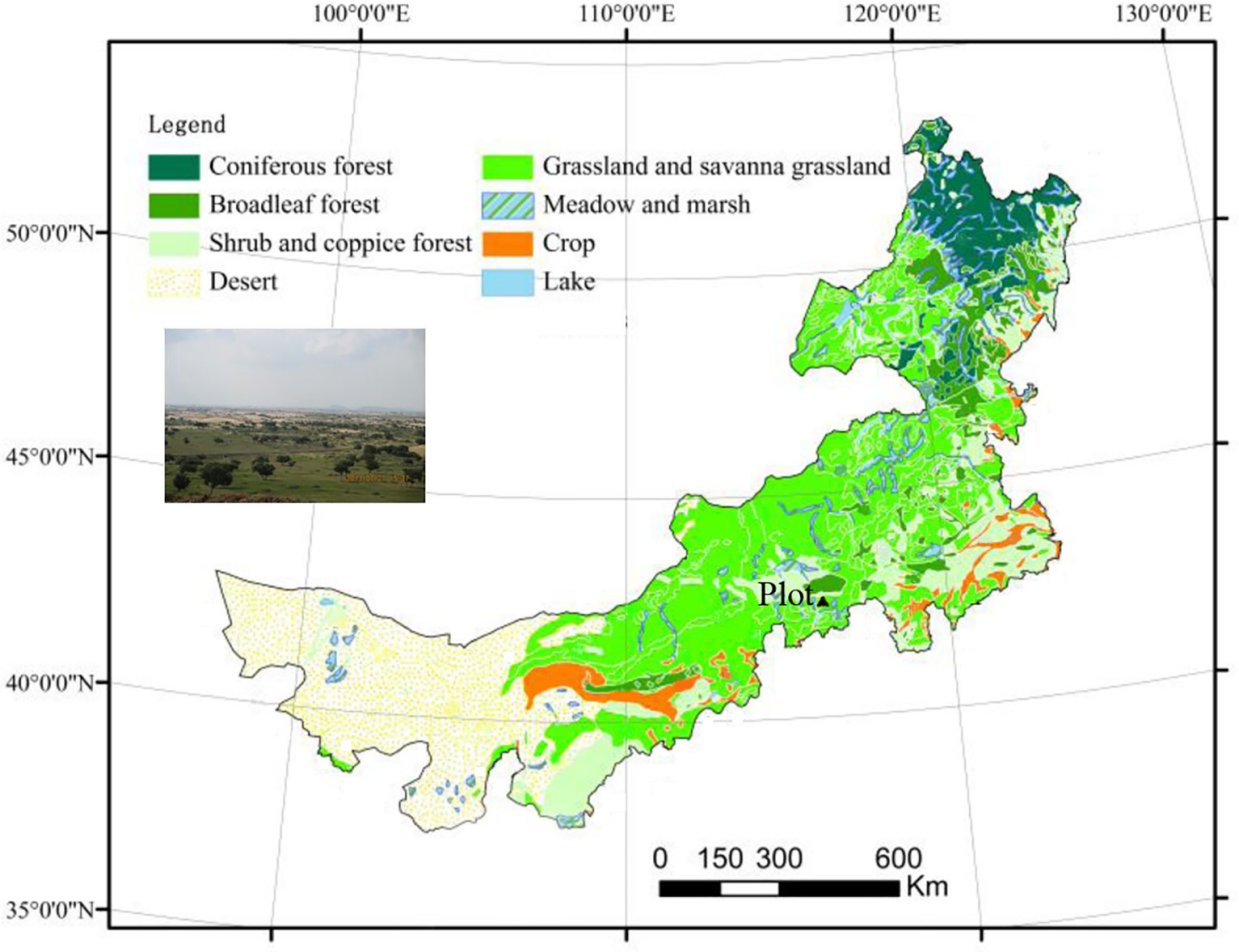
Map of the Otindag Sandy Land and the location site of the study region.

The study site is located in the Otindag Sandy Land (Fig 1). It is administered by the Sanggendalai township of the Xilinguole League, Inner Mongolia (115°16'E, 42°50'N). The elevation is approximately 1320 m. The local climate is continental, with hot summers, long and cold winters, and a mean annual precipitation of 250–350 mm, more than 70% of which occurs from June to August. The annual mean temperature is 1.7°C, the extreme minimum temperature is -38°C, and the annual accumulated temperature (≧10°C) is 2000°C. The frostless period is approximately 105 days, the annual sunshine duration is greater than 1000 h, the annual mean wind speed is 4 m/s, and the wind level exceeding a Beaufort scale value of 8 is 90 days per year. The main soil types are aeolian sandy soil with a mean depth of 200 cm and a calcic horizon occurring at a depth of 30–100 cm. This horizon is extremely hard and does not allow plant roots to penetrate it.

The local herders currently select alternating seasonal grazing (winter and spring) and mowing (autumn) regimes, whereas annual grazing was previously implemented. The area’s grazing livestock are primarily cattle. To prevent grassland degradation, grazing is prohibited during the growing season of the grasses (summer), and the herders mow grass to reserve food for animals in winter. Both grazing and mowing occur every year. No fire has happened in this study site.

### Study species

#### Trees

*U*. *pumila*: This tree can survive to an age of more than 40 years. The species can grow to a height of more than 10 m. *U*. *pumila* seeds are wind-dispersed and can be dispersed to a great distance from their parents during the windy season.

#### Shrubs

Xerophytic shrubs: *Caragana microphylla* Lam is a long-lived shrub with multiple stems bearing thorns and compound leaves. Flowering occurs in May and lasts approximately 20 days. Seeds ripen in July, and seed germination is triggered by summer rainfall. Shoots usually grow out at the end of the flowering period. *Spiraea aquilegifolia* Pall is a long-lived shrub. Flowering occurs in May-July, and seeding occurs in August-September; *Ribes diacanthum* is a long-lived shrub. Flowering occurs in May-June, and seeding occurs in July-August; *Salix linearitipularis* is a long lived shrub.

Mesophytic shrubs: *Betula fruticosa* Pall is a long-lived shrub. Seeds are dispersed by animals.

### Data collection

A fenced rangeland tract (approximately 50 ha), typical of *U*. *pumila*-dominated savanna and contracted by a herder family, was selected as the study site. The site was placed under alternative seasonal grazing and mowing management in approximately the year 2000. Seasonal grazing occurred in the winter and spring, and mowing was implemented in autumn with a small tractor mower. In August of 2014, 3 sample plots 100 m×100 m in size were established at fixed distances from the herder’s stationary site (numbered as plot 1, plot 2 and plot 3 from near to far). Plot 1 was 1 km distant from the herder’s stationary site, plot 2 was 2 km distant, and plot 3 was 3 km distant. All the 3 plots have basically identical natural conditions (such as soil types, geomorphology, rainfall). Each plot (100 m × 100 m) was divided into 400 contiguous 5 m × 5 m plots, and the name, diameter (DBH or collar diameter), height and spatial coordinates of all the woody plants (trees and shrubs) were recorded in each plot. All individual woody plants in the three plots were mapped using a total station transit (model GTS-3B, Topcon, Paramus, New Jersey, USA) with an accuracy of approximately 1 cm.

**Fig 2:**
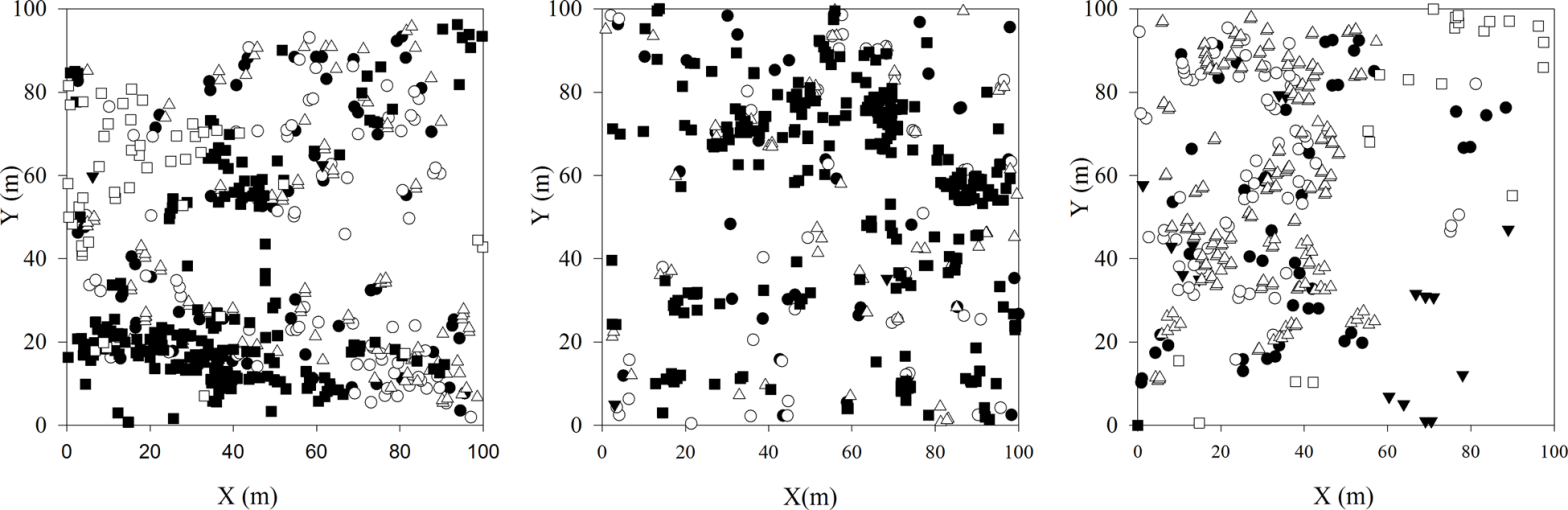
Distribution map of the woody plants in three surveyed plots. ● U. pumila-old trees,○U. pumila-medium trees,▼ U. pumila-juvenile trees, △ U. pumila-seedlings, ■ xerophytic shrubs, and □ mesophytic shrubs.

All the *U*. *pumila* trees were classified into 4 categories according to their DBH, namely, old adult trees (DBH ≥ 20 cm), medium adult trees (5 cm ≤ DBH < 20 cm), juvenile trees (DBH < 5 cm) and seedlings (without DBH), a classification that is consistent with previous research results [36]. Shrubs were also classified into two categories according to their ecophysiological traits[37], namely, xerophytes, including *Caragana microphylla* Lam, *Spiraea aquilegifolia* Pall, *Ribes diacanthum* and *Salix linearitipularis*, and mesophytes, including *Betula fruticosa* Pall. (Table 1; Fig 2). As few individuals in the juvenile tree category were found, this category was not included in the spatial pattern analysis.

**Table 1:** Classification of *U*. *pumila* trees and shrubs and their basic parameters.

*species*	*Category* *	*Plot 1*		*Plot 2*		*Plot 3*	
		*N*	*H*	*N*	*H*	*N*	*H*
*U*. *pumila trees*	*Old*	*40*	*7*.*4±0*.*94*	*63*	*7*.*3±1*.*53*	*47*	*7*.*6±1*.*23*
	*Medium*	*50*	*5*.*3±0*.*93*	*85*	*5*.*1±1*.*27*	*68*	*5*.*5±0*.*86*
	*Juvenile*	*2*	*1*.*7±0*.*30*	*2*	*2*.*4±0*.*22*	*16*	*2*.*3±0*.*30*
	*Seedling*	*53*	*0*.*23±0*.*16*	*108*	*0*.*28±0*.*22*	*214*	*0*.*30±0*.*21*
*xerophytic shrubs*	*CaraMicr*	*58*	*0*.*41±0*.*13*	*27*	*0*.*39±0*.*10*	*/*	*/*
	*SpirAqui*	*215*	*0*.*42±0*.*17*	*158*	*0*.*41±0*.*15*	*/*	*/*
	*RibeDiac*	*/*	*/*	*12*	*0*.*31±0*.*10*	*/*	*/*
	*SaliLine*	*/*	*/*	*17*	*2*.*0±0*.*21*	*/*	*/*
*mesophytic shrubs*	*BetuFrut*	*/*	*/*	*46*	*2*.*2±0*.*35*	*21*	*2*.*4±0*.*13*

* Latin name abbreviations: *CaraMicr- Caragana microphylla* Lam., *SpirAqui- Spiraea aquilegifolia* Pall, *RibeDiac- Ribes diacanthum*, *SaliLine- Salix linearitipularis*, *BetuFrut- Betula fruticosa* Pall.

### Data analysis

Ripley’s K-function and the pair-correlation g-function are common techniques for univariate and bivariate point-pattern analysis. The function K(r) is the expected number of points in a circle of radius r centered at an arbitrary point (which is not counted), divided by the intensity λ of the pattern.

The alternative pair correlation function g(r), which arises if the circles of Ripley’s K-function are replaced by rings, gives the expected number of points at distance r from an arbitrary point, divided by the intensity λ of the pattern. The function g(r) has the additional advantage that it is a probability density function with the interpretation of a neighborhood density, which is more intuitive than an accumulative measure[38]. Therefore, the pair-correlation function g(r) is especially sensitive to small-scale effects. In this study, we used univariate and bivariate pair correlation functions g(r) and g_12_(r) to quantify the spatial pattern of individual woody plants and interactions among them. For univariate analysis, the formula used[31, 39–41] is as follows:

$$g\left(t\right)=\frac{1}{2\pi t}\frac{A^{2}}{n^{{}_{2}}}\sum_{i=1}^{n}\sum_{\begin{array}{l} i=1\\ j\neq 1 \end{array}}^{n}w_{ij}^{-1}k_{h}\left(t-\left|x_{i}-x_{j}\right|\right),$$

where A is the plot area, n is the total number of plants and w_ij_ is a weighting factor correcting for edge effects. k_h_ is a kernel function, which is a weighting function applying maximum weight to point pairs with a distance exactly equal to t but incorporating point pairs at an approximate distance t with reduced weight. This weight falls to zero if the actual distance between the points differs from t more than h, the so-called bandwidth parameter. At a given distance r, values of g (r) > 1 indicate that interpoint distances around r are relatively more frequent than they would be under complete spatial randomness (CSR). If this is the case for small values of r, there is typically clustering. Conversely, values of g(r) < 1 indicate that interpoint distances around r are relatively less frequent than they would be under CSR. If this is the case for small values of r, the pattern shows regularity.

For bivariate analysis, the formula used[39–41] is as follows:

$$g_{12}\left(t\right)=\frac{1}{2\pi t}\frac{A^{2}}{n_{1}n_{2}}\sum_{i=1}^{n_{1}}\sum_{i=1}^{n_{2}}w_{ij}^{-1}k_{h}\left(t-\left|x_{i}-x_{j}\right|\right),$$

where x_i_, i = 1,…, n_1_, and y_j_, j = 1,…, n_2_ are the points of groups 1 and 2, respectively, with the same weights w_ij_ and kernel function k_h_ as above. At a given distance r, values of g_12_ (r) > 1 indicate that species 2 is positively associated with species 1 at distance r. Values of g_12_ (r) = 1 indicate that there is no interaction between species 1 and 2. Values of g_12_ (r) <1 indicate that species 2 is negatively associated with species 1.

The keys for successful application of the g function are the selection of an appropriate null model that responds to the specific biological question asked and the correct interpretation of a given departure of data from the null model [40]. In this study, we used the null model of complete spatial randomness (CSR) as a null hypothesis for the univariate analyses of three tree categories (old trees, medium trees and seedlings), and for two shrub categories (xerophytes, mesophytes).

For the bivariate analyses, two cases were considered. In the first case, the relationship between small and large trees was considered. Because large trees may impact the distribution pattern of small trees within their area of influence (competition), we conducted a bivariate g function analysis for these two size classes using both the toroidal shift and the antecedent condition null model options[40]. This tests whether the patterns of distribution of small and large trees were generated by independent processes. The antecedent condition model tests whether one pattern (small trees) is influenced by a second pattern (large trees), assessing whether there are more (or fewer) small trees in the neighborhood of large trees than expected under a random distribution of small trees [39]. The second case concerns the interaction between trees and shrubs. Because the spatial distributions of plants in plots seem to be affected significantly by drought stress and habitat heterogeneity (e.g., soil patch and microtopography), we examined the spatial association between the two species with the independent null model [40].

To assess the significance of the test function under the null model, we generated an approximate (two-sided) 95% simulation envelope by calculating for each distance r the 5th lowest and highest values of the summary statistic from 199 Monte Carlo simulations of the null model. All analyses were performed using the software Programita for point pattern analysis [40].

## Results

### Univariate analysis of *U*. *pumila* trees

**Fig 3:**
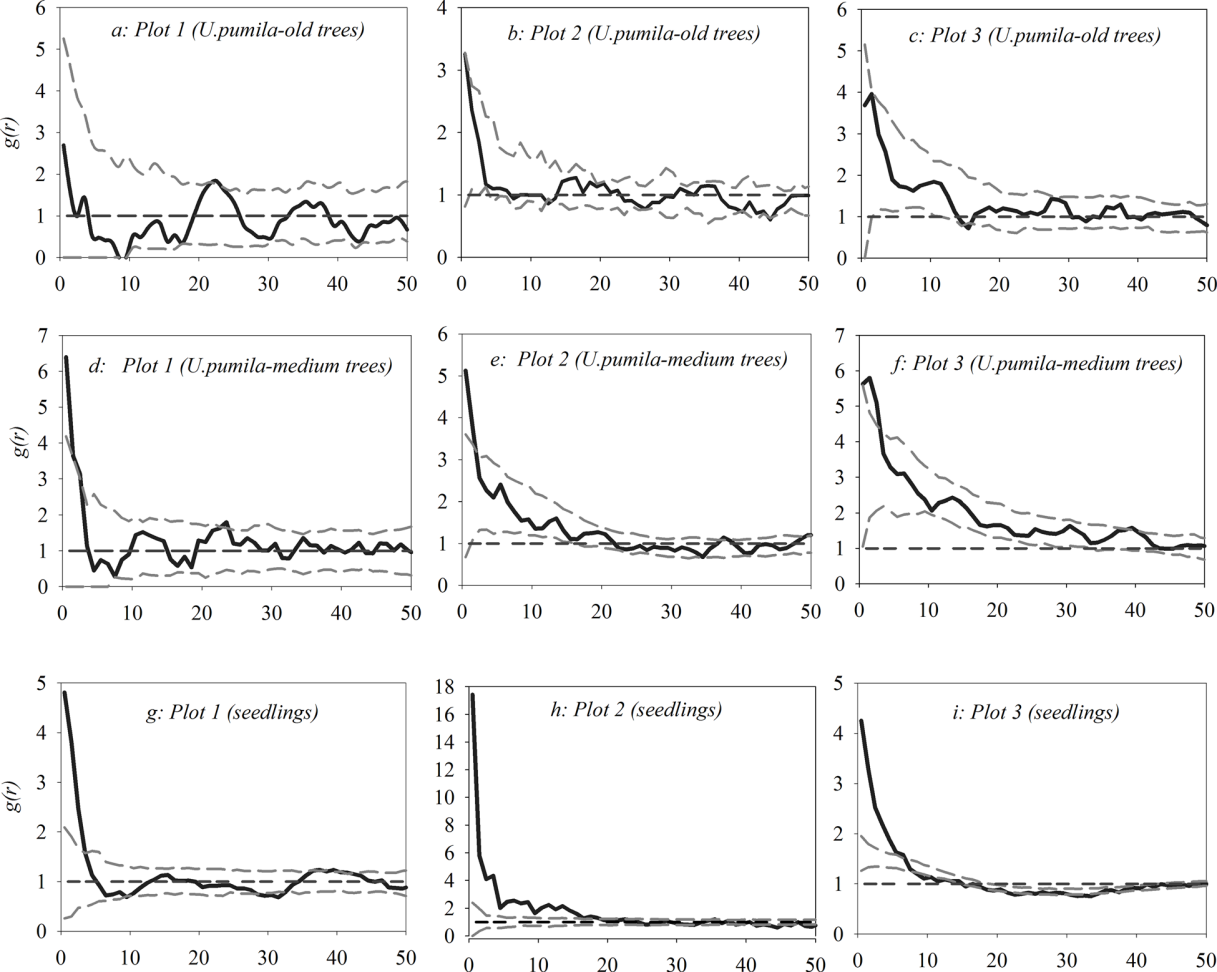
Univariate spatial patterns of three *U*. *pumila* tree categories with the null model of CSR.

As shown in Fig 3(see S1 Fig), old *U*. *pumila* trees were randomly distributed at all scales within all 3 plots, medium *U*. *pumila* trees exhibited a significant aggregated trend within 0–2.5 m in all 3 plots, and seedlings were significantly aggregated within 0–3.5 m in plot 1, within a 0–18.5 m scale in plot 2 and within 0–6.5 m in plot 3. All 3 tree categories were randomly distributed at other scales within all 3 plots.

### The univariate analysis of shrubs

**Fig 4:**
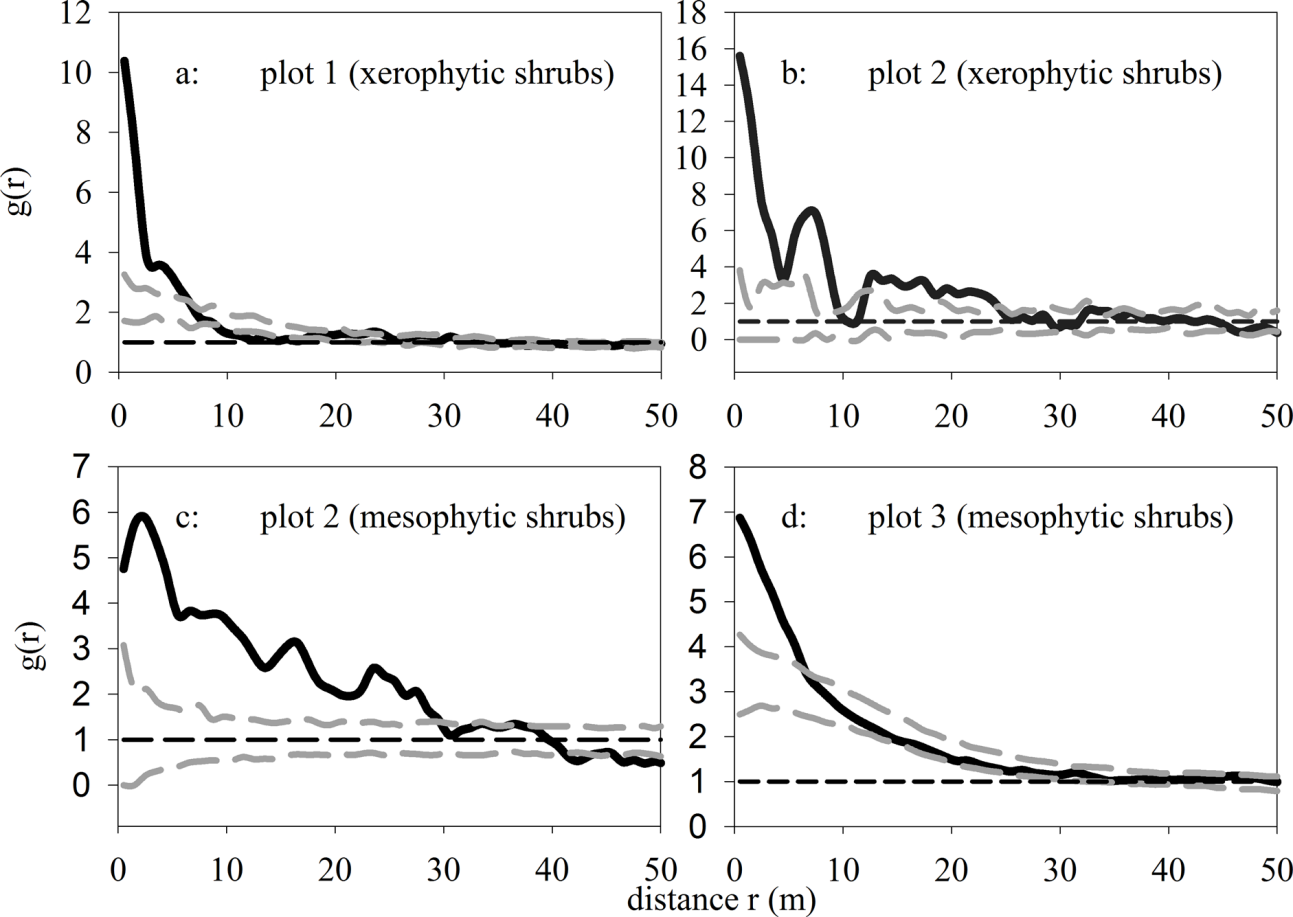
Univariate spatial patterns of two shrub categories with the null model of CSR.

As shown in Fig 4(see S2 Fig), xerophytic shrubs were significantly aggregated within 0–5.5 m in plot 1, within 0–6.5 m in plot 2, and mesophytic shrubs showed a tendency for a significant or insignificant aggregation within 0–23.5 m in plot 2 and within 0–29.5 m in plot 3. The two shrub categories were randomly distributed at other scales within all 3 plots.

### Bivariate spatial analysis of three *U*. *pumila* tree categories

**Fig 5:**
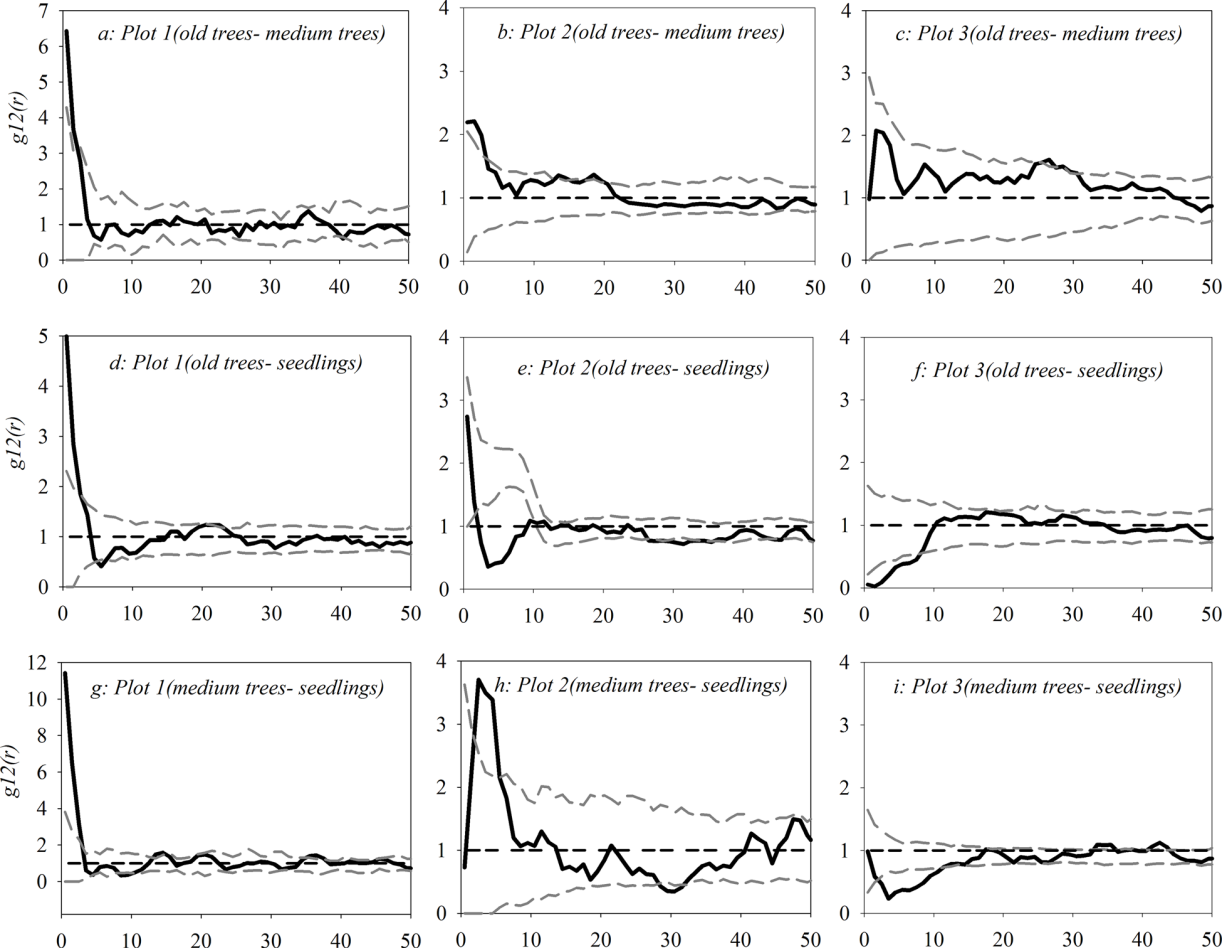
Bivariate spatial association between three *U*. *pumila* tree categories with both the toroidal shift and antecedent condition null models.

As shown in Fig 5(see S3 Fig), old trees and medium trees showed a significant positive correlation within 0–4 m in plot 1 and plot 2 and weak positive correlation within 4–20 m and within 0–45 m in plot 2 and plot 3, respectively. Old trees and seedlings showed a significant positive correlation within 0–2 m in plot 1 and a significant negative correlation within 3–10 m and within 0–10 m in plot 2 and plot 3, respectively. However, they did not show a significant correlation at other scales. Medium trees and seedlings showed a significantly positive correlation within 0–3 m and within a 1–6 m scale in plot 1 and plot 2 and a significant negative correlation within 1–11 m in plot 3.

### Bivariate spatial analysis of *U*. *pumila* tree categories and shrub categories

**Fig 6:**
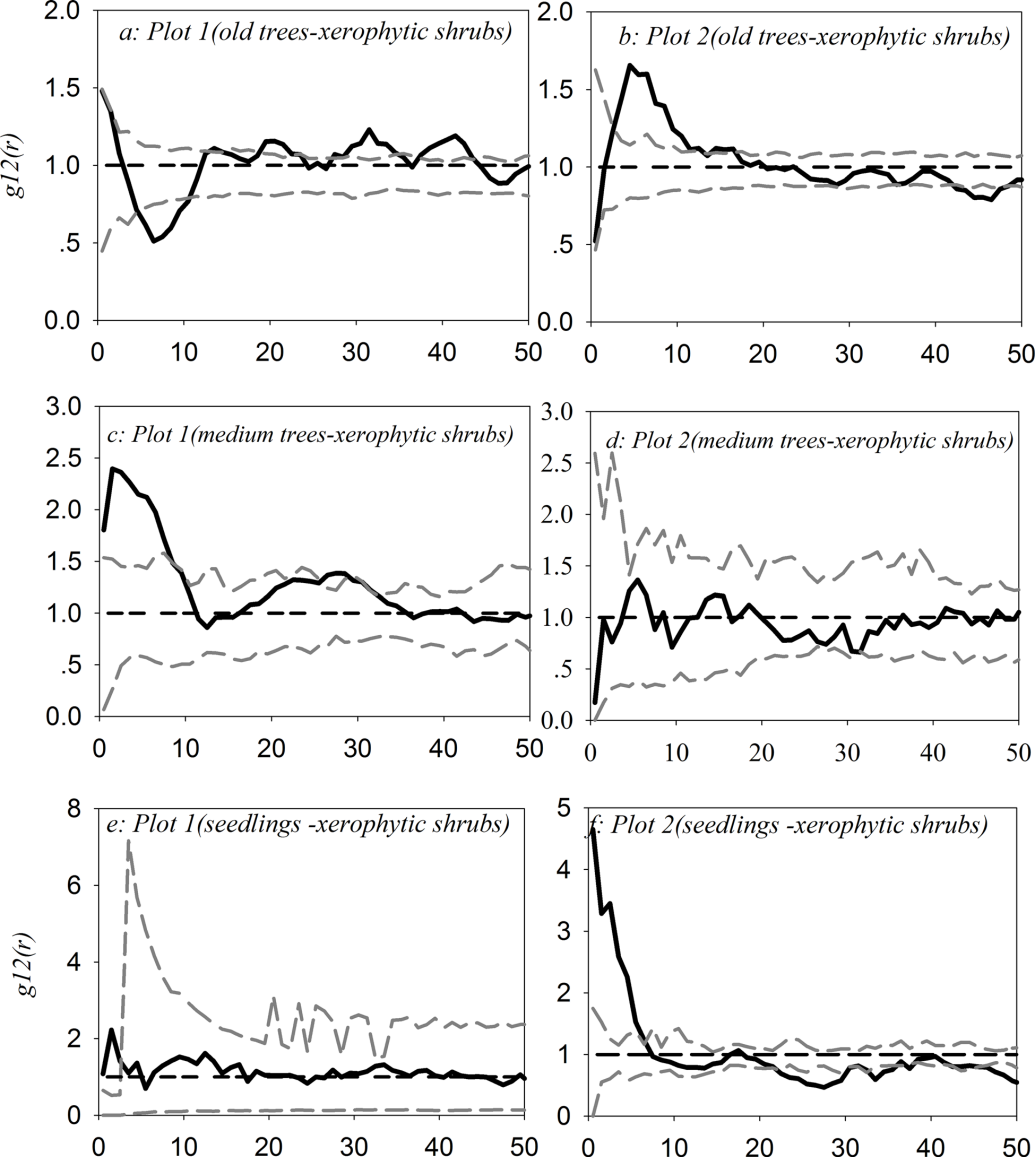
Bivariate spatial association between *U*. *pumila* trees and xerophytic shrubs in plot 1 and plot 2 with the independent null model.

As shown in Fig 6(see S4 Fig), old trees and xerophytic shrubs had a significant negative correlation within 5–10 m and a significant positive correlation within 3–11 m in plot 1 and plot 2, respectively; medium trees and xerophytic shrubs had a significant positive correlation within 0–10 m only in plot 1, and seedlings and xerophytic shrubs had a significant positive correlation within 0–2 m and 0–7 m in plot 1 and plot 2, respectively.

**Fig 7:**
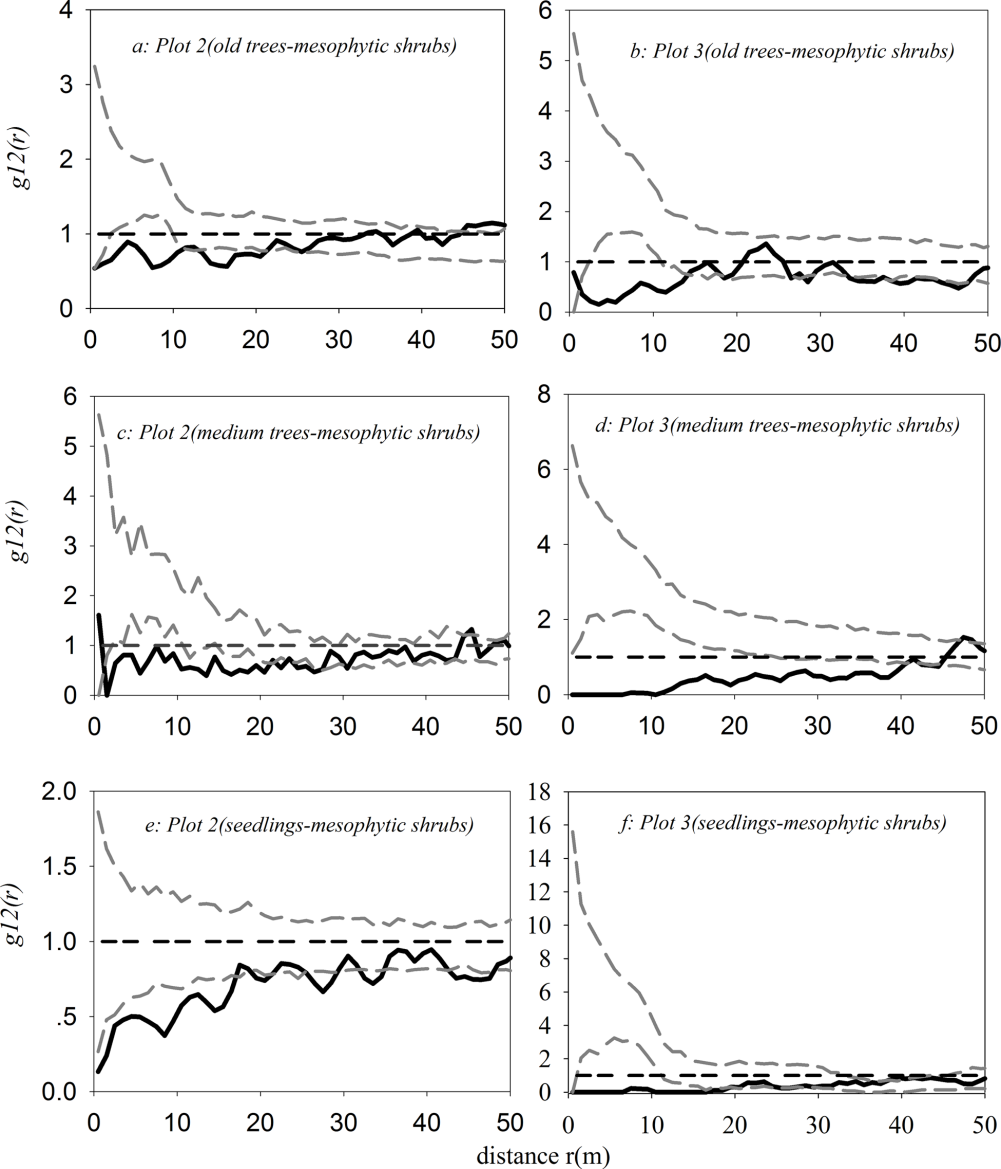
Bivariate spatial association between *U*. *pumila* trees and mesophytic shrubs in plot 2 and plot 3 with the independent null model.

As shown in Fig 7(see S4 Fig), old trees and mesophytic shrubs had a significant negative correlation within 0–20 m and within 1–15 m in plot 2 and plot 3, respectively. Medium trees and mesophytic shrubs had a significant negative correlation within 0–20 m and 0–40 m in plot 2 and plot 3, and seedlings and mesophytic shrubs had a significant negative correlation within 0–20 m and 0–19 m in plot 2 and plot 3, respectively.

## Discussion

### Spatial patterns of woody plants and their mechanism of formation

#### *U*. *pumila* trees

**Fig 8:**
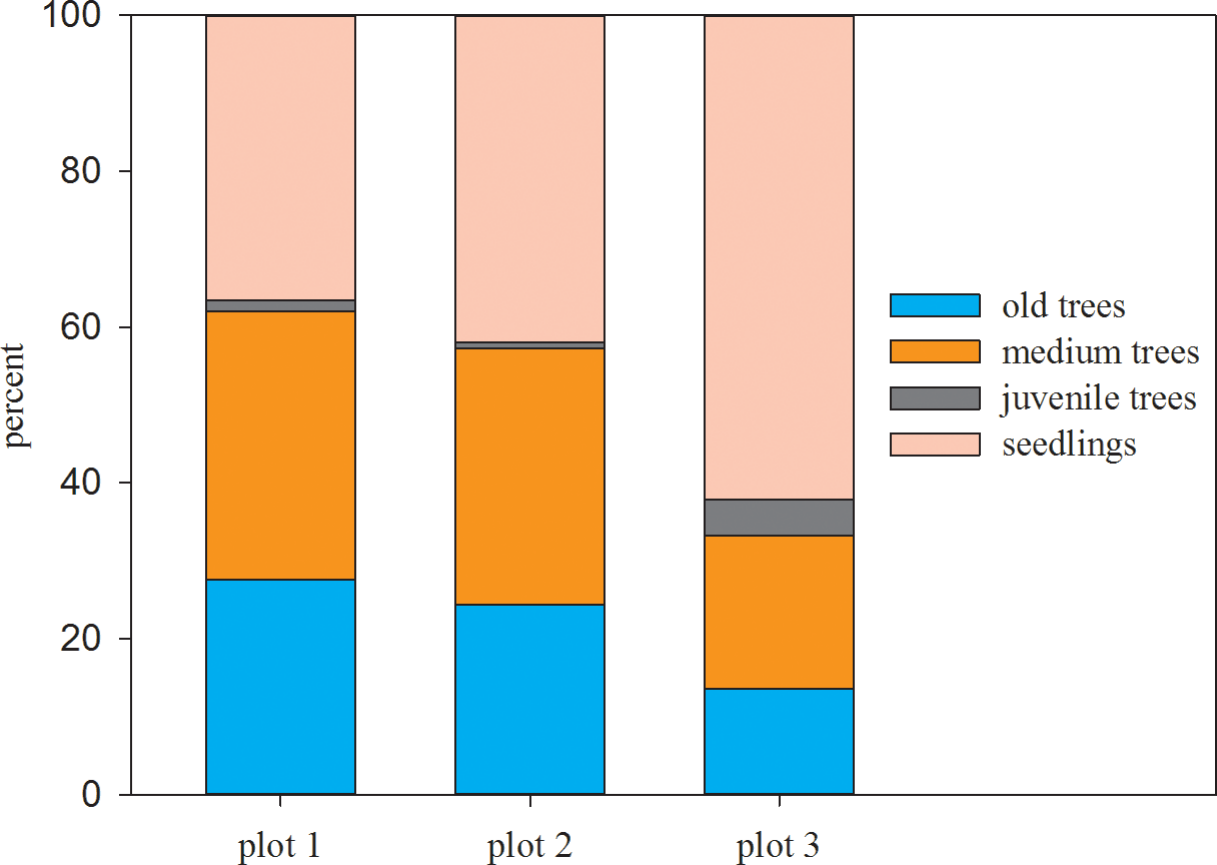
The proportion of different age-class categories of a *U*. *pumila* tree.

Tree species show different spatial patterns in their different growth stages and among the different age classes. These patterns are closely related to the self-thinning process, to the disturbance pattern(such as fire, grazing and so on) and to environmental change[42]. Analyzing the spatial patterns of different growth stages can yield information about the regeneration of new individuals, the morality of adults, and the overall demographics of a population following natural or human disturbance[43]. In general, young individuals aggregate, and adult individuals are randomly or uniformly distributed within a species [44]. In this study, old trees showed a random distribution, and medium *U*. *pumila* trees showed an aggregated distribution (0–2.5 m) in the smaller scales and a random distribution in the larger scales among all 3 plots. These patterns may result from the patchiness of water and soil resources at smaller scales in a semi-arid area[45]; however, these adult trees have formed a relatively stable structure in this type of temperate savanna despite differences in grazing intensity among the 3 plots, and their existence does not reduce the productivity of the herbaceous communities under their canopy [46]. These trees can, in turn, provide a shady resting habitat for domestic livestock during hot grazing seasons [47]. According to the results of Peng [36], two categories of old and medium adult trees have grown for more than 15 years; that is, the occurrence of these individuals, prior to the implementation of the alternative seasonal grazing-mowing regime, is a result of interactions among year-round free grazing and abiotic factors (such as climate, soil and microgeomorphology)[48]. There are fewer juvenile trees in all 3 plots, therefore their spatial pattern have not been analysed in this study (Table 1; Fig 8 and S5 Fig), the individual age of the juvenile tree category is less than 15 years old, which coincides with the implementation period of the alternative seasonal grazing-mowing regime to a great extent; it can be deduced that the majority of one-year or multiyear seedlings that remained and grew from juveniles to adults[49, 50] during the year-round free grazing procedure due to selective feeding of livestock, might be harvested by the nonselective mowing activity in autumn[51–54]. This deduction can better explain why numerous seedlings (germinated mostly in the current year) existed in aggregations in our August investigation. This phenomenon interrupted the chain of normal regeneration of *U*. *pumila* trees. This severe human-caused disturbance, which may eventually result in the breakdown of the *U*. *pumila* tree community in the savanna ecosystem.

#### Shrubs

In arid and semi-arid systems, Shrubs layers are often dominant [55–57], species rich, and are planted for revegetation in bare sand or less-covered soil surfaces in arid and semi-arid areas of China [58–60]. However, shrub encroachment into rangeland has been considered an obvious degradation indicator around the world for more than 20 years[61–65], despite some arguments appearing in recent years[66, 67]. In this study, shrubs occurred in the *U*. *pumila*-dominated savanna along the grazing gradient as xerophytic shrubs only in plot 1, as both xerophytic shrubs and mesophytic shrubs in plot 2, and as mesophytic shrubs only in plot 3. Additionally, the xerophytic shrubs showed significant aggregations at a smaller scale (0–8 m) in plot 1 and plot 2, while the mesophytic shrubs showed significant or insignificant aggregations at a larger scale (0–25 m) in plot 2 and plot 3. We are not sure when these shrubs encroached on the savanna, whether before or after the implementation of the new management regime (alternative seasonal grazing-mowing regime), but it can be deduced from related studies that their encroachment resulted from the grazing pressure on the one hand and from long-term climate change on the other hand [68–71]. Mowing operations can obviously cut shrub saplings and seedlings while also cutting *U*. *pumila* seedlings, as noted above, but the sprouting traits of some shrubs can facilitate their existence in a human-disturbed habitat [72–74]. Based on this phenomenon, we may deduce that *Betula fruticosa*, the only mesophytic and nonsprouting shrub in the study site, might have been established before the implementation of the new management regime, while most of the observed xerophytic shrubs with sprouting traits might have encroached on the savanna after the implementation of the new management regime.

### Spatial interactions among woody plants

Among these three tree categories, there was a relatively weak positive correlation between old and medium trees in all 3 plots. Between the medium trees and seedlings, there was significant facilitation on a smaller scale in plot 1 and plot 2, which may result from wind-dispersed seed deposition around medium trees and better micro-habitat provision for seed germination and seedling growth by medium trees[75, 76]. In plot 3, significant competition at the 1–11 m scale occurred between medium trees and seedlings. This pattern may be the result of the occurrence of more seedlings around the medium trees due to the occurrence of less livestock trampling, associated with low grazing pressure [77–79].

Except in the case of the comparison between old trees and xerophytic shrubs in plot 1, facilitative relationships were significant between medium trees or seedlings and xerophytic shrubs in plot 1 and plot 2 (Fig 6). Significant competitive relationships were found among old trees, medium trees or seedling and mesophytic shrubs in plot 2 and plot 3 (Fig 7). These findings may esult entirely from the exploitation of soil and water by plants with different ecophysiological traits [80–82]. For the former, medium trees may act as nurse plants for xerophytic shrubs and seedlings, and xerophytic shrubs may also act as nurse plants for seedlings, in agreement with the “nurse plant syndrome” [83–86]; for the latter, their similar ecophysiological traits require them to maintain competitive relationships under the influence of climate change [87–89].

In conclusion, the implementation of a new management regime resulted in xerophytic shrub encroachment or existence, which may result from a dual effect of seasonal grazing and climate change, and broke the chain of normal succession of a *U*. *pumila* tree community in this typical temperate savanna ecosystem. Clearly, a new, commonly used (active or passive) management regime was used to replace the older traditional management regime. The new management regime might eventually result in the degradation of original tree-dominated savanna to xerophytic shrub-dominated savanna.

More field experiments should be undertaken to reveal underlying ecological processes (e.g. changes of soil and water) that gave rise to plant spatial pattern and to prove our deduction from pattern, and a more scientific and effective management regime should be studied to maintain the stability and sustainability of this typical tree-dominated savanna ecosystem in the present and near future.

## Supporting Information

S1 Fig This dataset contains a univariate analysis of the *U*. *pumila* tree and of shrubs. (XLSX)

S2 Fig This dataset contains a univariate analysis of shrubs. (XLSX)

S3 Fig This dataset contains a bivariate spatial analysis including three *U*. *pumila* tree categories. (XLSX)

S4 Fig This dataset contains a bivariate spatial analysis including *U*. *pumila* tree and shrubs. (XLSX)

S5 Fig This dataset contains the proportion of different age-class categories of *U*. *pumila* trees. (XLS)

## References

[1] Williams DM. Beyond great walls: environment, identity, and development on the Chinese grasslands of inner Mongolia: Stanford University Press; 2002.

[2] Xie Y, Li W. WHY DO HERDERS INSIST ON" OTOR?" MAINTAINING MOBILITY IN INNER MONGOLIA. Nomadic Peoples. 2008:35–52.

[3] Werger MJ, van Staalduinen MA. Eurasian steppes Ecological problems and livelihoods in a changing world: Springer Science & Business Media; 2012.

[4] Thwaites R, de Lacy T, Hong LY, Hua LX. Property rights, social change, and grassland degradation in Xilingol Biosphere Reserve, Inner Mongolia, China. Society & Natural Resources. 1998;11(4):319–38.

[5] Ho P. Rangeland degradation in north China revisited? A preliminary statistical analysis to validate non-equilibrium range ecology. Journal of Development Studies. 2001;37(3):99–133.

[6] Tong C, Wu J, Yong S-p, Yang J, Yong W. A landscape-scale assessment of steppe degradation in the Xilin River Basin, Inner Mongolia, China. Journal of Arid Environments. 2004;59(1):133–49.

[7] Li WJ, Ali SH, Zhang Q. Property rights and grassland degradation: A study of the Xilingol Pasture, Inner Mongolia, China. Journal of Environmental Management. 2007;85(2):461–70. 17129663 10.1016/j.jenvman.2006.10.010

[8] Zhang MA, Borjigin E, Zhang H. Mongolian nomadic culture and ecological culture: On the ecological reconstruction in the agro-pastoral mosaic zone in Northern China. Ecological Economics. 2007;62(1):19–26.

[9] Squires VR. Rangeland degradation and recovery in China's pastoral lands: CABI; 2009.

[10] Li A, Wu J, Huang J. Distinguishing between human-induced and climate-driven vegetation changes: a critical application of RESTREND in inner Mongolia. Landscape ecology. 2012;27(7):969–82.

[11] Li X-Y, Zhang S-Y, Peng H-Y, Hu X, Ma Y-J. Soil water and temperature dynamics in shrub-encroached grasslands and climatic implications: results from Inner Mongolia steppe ecosystem of north China. Agricultural and forest meteorology. 2013;171:20–30.

[12] Peng H-Y, Li X-Y, Li G-Y, Zhang Z-H, Zhang S-Y, Li L, et al Shrub encroachment with increasing anthropogenic disturbance in the semiarid Inner Mongolian grasslands of China. Catena. 2013;109:39–48.

[13] Hilbig W. vegetation of Mongolia: SPB Academic Pubishing; 1995.

[14] Fang J, Wang Z, Tang Z. Atlas of woody plants in China: distribution and climate: Springer Science & Business Media; 2011.

[15] Dunn CP. The elms: Breeding, conservation, and disease management: Springer Science & Business Media; 2000.

[16] Zhang X. Vegetation of China and its geographic patterns. Beijing: Geological publishing House; 2007.

[17] Ci Longjun YX. Desertification and its control in China. Beijing: Higher Education Press and Heidelberg: Springer-Verlag; 2010 513 p.

[18] Liu L, Wang H, Lin C, Wang D. Vegetation and community changes of elm (Ulmus pumila) woodlands in Northeastern China in 1983–2011. Chinese geographical science. 2013;23(3):321–30.

[19] Su H, Li Y, Liu W, Xu H, Sun OJ. Changes in water use with growth in Ulmus pumila in semiarid sandy land of northern China. Trees. 2014;28(1):41–52.

[20] Jiuru X, Shiming C, Shouyi Z. Collection, conservation and propagation techniques of the old elm (Ulmus pumila L.) tree resources in Ke'erqin sand land. Journal beijing forestry university-chinese edition. 2001;23(5):75–8.

[21] Shi L, Zhang Z, Zhang C, Zhang J. Effects of sand burial on survival, growth, gas exchange and biomass allocation of Ulmus pumila seedlings in the Hunshandak Sandland, China. Annals of Botany. 2004;94(4):553–60. 15329332 10.1093/aob/mch174 PMC4242228

[22] Wang X, Hu C, Li G, Zuo H. Analysis of the factors affecting seed disperal and seedling survival rate of Ulmus pumila in the Otindag sandy land. Arid Zone Research. 2011;28:542–7.

[23] Jiang D, Tang Y, Busso CA. Effects of vegetation cover on recruitment of Ulmus pumila L. in Horqin Sandy Land, northeastern China. Journal of Arid Land. 2014;6(3):343–51.

[24] Guo K, Liu H. A comparative researches on the development of elm seedings in four habitats in the Hunshandak Sandland, Inner Mongolia, Chian. Acta Ecologica Sinica. 2003;24(9):2024–8.

[25] Wesche K, Walther D, Von Wehrden H, Hensen I. Trees in the desert: Reproduction and genetic structure of fragmented Ulmus pumila forests in Mongolian drylands. Flora-Morphology, Distribution, Functional Ecology of Plants. 2011;206(2):91–9.

[26] Dale MRT. Spatial pattern analysis in plant ecology. Ecology. 1999;88:366–70.

[27] Wiegand T, Moloney KA. Handbook of spatial point-pattern analysis in ecology: CRC Press; 2013.

[28] Fangliang H, Legendre P, LaFrankie JV. Distribution patterns of tree species in a Malaysian tropical rain forest. Journal of Vegetation Science. 1997:105–14.

[29] Druckenbrod DL, Shugart HH, Davies I. Spatial pattern and process in forest stands within the Virginia piedmont. Journal of Vegetation Science. 2005;16(1):37–48.

[30] Nathan R. Long-distance dispersal of plants. Science. 2006;313(5788):786–8. 16902126 10.1126/science.1124975

[31] Condit R, Ashton PS, Baker P, Bunyavejchewin S, Gunatilleke S, Gunatilleke N, et al Spatial patterns in the distribution of tropical tree species. Science. 2000;288(5470):1414–8. 10827950 10.1126/science.288.5470.1414

[32] Bossdorf O, Schurr F, Schumacher J. Spatial patterns of plant association in grazed and ungrazed shrublands in the semi-arid Karoo, South Africa. Journal of Vegetation Science. 2000:253–8.

[33] Schurr FM, Bossdorf O, Milton SJ, Schumacher J. Spatial pattern formation in semi-arid shrubland: a priori predicted versus observed pattern characteristics. Plant Ecology. 2004;173(2):271–82.

[34] Wang Y, Yang X, Shi Z. The formation of the patterns of desert shrub communities on the western Ordos Plateau, China: the roles of seed dispersal and sand burial. PloS one. 2013;8(7):e69970 10.1371/journal.pone.0069970 23922877 PMC3724907

[35] Wu B, Yang H. Spatial patterns and natural recruitment of native shrubs in a semi-arid sandy land. PloS one. 2013;8. 10.1371/journal.pone.0058331 PMC3591353 23505489

[36] Yu P. Restoring degraded ecosystem in Hunshandak Sand Land through nature reserve. Beijing: Chinese Academy of Science; 2005.

[37] Wu Z, Raven PH, Hong D. (Eds) Flora of China Beijing: Science Press and St. Louis: Missouri Botanical Garden Press;2013 (online: http://foc.eflora.cn/)

[38] Stoyan D, Stoyan H. Improving ratio estimators of second order point process characteristics. Scandinavian Journal of Statistics. 2000:641–56.

[39] Stoyan D, Stoyan H. Fractals, random shapes, and point fields: methods of geometrical statistics: Wiley Chichester; 1994.

[40] Wiegand T, A Moloney K. Rings, circles, and null—models for point pattern analysis in ecology. Oikos. 2004;104(2):209–29.

[41] Illian J, Penttinen A, Stoyan H, Stoyan D. Statistical analysis and modelling of spatial point patterns: John Wiley & Sons; 2008.

[42] Greig-Smith P. Quantitative plant ecology: Univ of California Press; 1983.

[43] Liu G, Ding Yi, Zang Runguo. Distribution patterns of Picea schrenk iana var. tianschanica population in Tianshan Mountains. Chinese Journal of Applied Ecology. 2011;22(1):9–13. 21548281

[44] Zhang J, Hao Z, Song B, Ye J, Li B, Yao X. Spatial distribution patterns and associations of Pinus koraiensis and Tilia amurensis in broad-leaved Korean pine mixed forest in Changbai Mountains.The journal of applied ecology. 2007;18(8):1681–7. 17974229

[45] Gutterman Y. Regeneration of plants in arid ecosystems resulting from patch disturbance: Springer Science & Business Media; 2001.

[46] Yang H, Chu J, Lu Q, Gao T. Relationships of native trees with grasses in a temperate, semi—arid sandy ecosystem of northern China. Applied vegetation science. 2014;17(2):338–45.

[47] Breman H, Kessler J. Woody plants in agro-ecosystems of semi-arid regions: with an emphasis on the Sahelian countries: Springer Science & Business Media; 2012.

[48] Vallentine JF. Grazing management: Elsevier; 2000.

[49] Manly B, McDonald L, Thomas D, McDonald TL, Erickson WP. Resource selection by animals: statistical design and analysis for field studies: Springer Science & Business Media; 2007.

[50] Gordon IJ, Prins HH. The ecology of browsing and grazing: Springer; 2008.

[51] Davies K, Bates J, Nafus A. Vegetation response to mowing dense mountain big sagebrush stands. Rangeland Ecology & Management. 2012;65(3):268–76.

[52] Shao C, Chen J, Li L, Zhang L. Ecosystem responses to mowing manipulations in an arid Inner Mongolia steppe: an energy perspective. Journal of Arid Environments. 2012;82:1–10.

[53] Yang H, Jiang L, Li L, Li A, Wu M, Wan S. Diversity—dependent stability under mowing and nutrient addition: evidence from a 7—year grassland experiment. Ecology Letters. 2012;15(6):619–26. 10.1111/j.1461-0248.2012.01778.x 22487498

[54] Spencer D, Enloe S, Pitcairn M, Ditomaso J. Impacts of mowing and bud destruction on Centaurea solstitialis growth, flowering, root dynamics and soil moisture. Weed research. 2014;54(2):140–50.

[55] NE W. Temperate deserts and semi-deserts. Amsterdam: Elsevier Scientific Publishing Company; 1983.

[56] Xin-Rong L. Study on shrub community diversity of Ordos Plateau, Inner Mongolia, northern China. Journal of Arid Environments. 2001;47(3):271–9.

[57] Garner W, Steinberger Y. A proposed mechanism for the formation of fertile islands in the desert ecosystem. Journal of arid Environments. 1989;16(3):257–62.

[58] Su YZ, lin Zhao H. Soil properties and plant species in an age sequence of Caragana microphylla plantations in the Horqin Sandy Land, north China. Ecological Engineering. 2003;20(3):223–35.

[59] Zhang T-H, Yong-Zhong S, Jian-Yuan C, Zhang Z-H, Chang X-X. A leguminous shrub (Caragana microphylla) in semiarid sandy soils of north China. Pedosphere. 2006;16(3):319–25.

[60] Li Y, Chen J, Cui J, Zhao X, Zhang T. Nutrient resorption in Caragana microphylla along a chronosequence of plantations: implications for desertified land restoration in North China. Ecological Engineering. 2013;53:299–305.

[61] Van Auken OW. Shrub invasions of North American semiarid grasslands. Annual Review of Ecology and Systematics. 2000:197–215.

[62] Jackson RB, Banner JL, Jobbágy EG, Pockman WT, Wall DH. Ecosystem carbon loss with woody plant invasion of grasslands. Nature. 2002;418(6898):623–6. 12167857 10.1038/nature00910

[63] Cabral A, Miguel J, Rescia A, Schmitz M, Pineda F. Shrub encroachment in Argentinean savannas. Journal of Vegetation Science. 2003;14(2):145–52.

[64] D'Odorico P, Okin GS, Bestelmeyer BT. A synthetic review of feedbacks and drivers of shrub encroachment in arid grasslands. Ecohydrology. 2012;5(5):520–30.

[65] Ratajczak Z, Nippert JB, Collins SL. Woody encroachment decreases diversity across North American grasslands and savannas. Ecology. 2012;93(4):697–703. 22690619 10.1890/11-1199.1

[66] Maestre FT, Bowker MA, Puche MD, Belén Hinojosa M, Martínez I, García—Palacios P, et al Shrub encroachment can reverse desertification in semi—arid Mediterranean grasslands. Ecology letters. 2009;12(9):930–41. 10.1111/j.1461-0248.2009.01352.x 19638041

[67] Maestre FT, Puche MD, Guerrero C, Escudero A. Shrub encroachment does not reduce the activity of some soil enzymes in Mediterranean semiarid grasslands. Soil Biology and Biochemistry. 2011;43(8):1746–9.

[68] Zheng Y, Xie Z, Robert C, Jiang L, Shimizu H. Did climate drive ecosystem change and induce desertification in Otindag sandy land, China over the past 40 years? Journal of Arid Environments. 2006;64(3):523–41.

[69] D'Odorico P, Fuentes JD, Pockman WT, Collins SL, He Y, Medeiros JS, et al Positive feedback between microclimate and shrub encroachment in the northern Chihuahuan desert. Ecosphere. 2010;1(6):art17.

[70] Cipriotti PA, Aguiar MR. Direct and indirect effects of grazing constrain shrub encroachment in semi—arid Patagonian steppes. Applied Vegetation Science. 2012;15(1):35–47.

[71] da Silveira Pontes L, Magda D, Jarry M, Gleizes B, Agreil C. Shrub encroachment control by browsing: Targeting the right demographic process. Acta Oecologica. 2012;45:25–30.

[72] Collins SL, Knapp AK, Briggs JM, Blair JM, Steinauer EM. Modulation of diversity by grazing and mowing in native tallgrass prairie. Science. 1998;280(5364):745–7. 9563952 10.1126/science.280.5364.745

[73] Calvo L, Tárrega R, Luis Ed, Valbuena L, Marcos E. Recovery after experimental cutting and burning in three shrub communities with different dominant species. Plant Ecology. 2005;180(2):175–85.

[74] Quero JL, Maestre FT, Ochoa V, García-Gómez M, Delgado-Baquerizo M. On the importance of shrub encroachment by sprouters, climate, species richness and anthropic factors for ecosystem multifunctionality in semi-arid Mediterranean ecosystems. Ecosystems. 2013;16(7):1248–61. 27330403 10.1007/-s10021-013-9683-y PMC4912035

[75] Brooker RW, Maestre FT, Callaway RM, Lortie CL, Cavieres LA, Kunstler G, et al Facilitation in plant communities: the past, the present, and the future. Journal of Ecology. 2008;96(1):18–34.

[76] Callaway RM. Positive interactions and community organization Positive Interactions and Interdependence in Plant Communities: Springer; 2007.

[77] Watt T, Gibson C. The effects of sheep grazing on seedling establishment and survival in grassland. Vegetatio. 1988;78(1–2):91–8.

[78] Hall LM, George MR, McCreary DD, Adams TE. Effects of cattle grazing on blue oak seedling damage and survival. Journal of Range Management. 1992:503–6.

[79] Zida D, Tigabu M, Sawadogo L, Tiveau D, Odén PC. Long—term effects of prescribed early fire, grazing and selective tree cutting on seedling populations in the Sudanian savanna of Burkina Faso. African Journal of Ecology. 2009;47(1):97–108.

[80] Roy J, Caldwell MM, Pearce RP. Exploitation of environmental heterogeneity by plants: ecophysiological processes above-and belowground: Academic Press; 2012.

[81] Barnes PW, Archer S. Tree—shrub interactions in a subtropical savanna parkland: competition or facilitation? Journal of Vegetation Science. 1999;10(4):525–36.

[82] Zou C, Barnes P, Archer S, McMurtry C. Soil moisture redistribution as a mechanism of facilitation in savanna tree—shrub clusters. Oecologia. 2005;145(1):32–40. 15942764 10.1007/s00442-005-0110-8

[83] Padilla FM, Pugnaire FI. The role of nurse plants in the restoration of degraded environments. Frontiers in Ecology and the Environment. 2006;4(4):196–202.

[84] Flores J, Jurado E. Are nurse—protégé interactions more common among plants from arid environments? Journal of Vegetation Science. 2003;14(6):911–6.

[85] Smit C, Ruifrok JL. From protege to nurse plant: establishment of thorny shrubs in grazed temperate woodlands. Journal of Vegetation Science. 2011;22(3):377–86.

[86] Gómez-Aparicio L, Zamora R, Gómez JM, Hódar JA, Castro J, Baraza E. Applying plant facilitation to forest restoration: a meta-analysis of the use of shrubs as nurse plants. Ecological Applications. 2004;14(4):1128–38.

[87] Grace J. Perspectives on plant competition: Elsevier; 2012.

[88] Dormann C. Herbivore—Mediated Competition between Defended and Undefended Plant Species: A Model to Investigate Consequences of Climate Change. Plant Biology. 2002;4(5):647–54.

[89] Schwinning S, Kelly CK. Plant competition, temporal niches and implications for productivity and adaptability to climate change in water—limited environments. Functional Ecology. 2013;27(4):886–97.

